# The role of remote ischemic preconditioning in the treatment of atherosclerotic diseases

**DOI:** 10.1002/brb3.161

**Published:** 2013-08-30

**Authors:** Spyros N Vasdekis, Dimitrios Athanasiadis, Andreas Lazaris, Georgios Martikos, Aristeidis H Katsanos, Georgios Tsivgoulis, Anastasios Machairas, Theodoros Liakakos

**Affiliations:** 1 Vascular Unit, Third Department of Surgery, School of Medicine Athens, University of AthensAthens, Greece; 2 Third Department of Surgery, University of Athens, School of MedicineAthens, Greece; 3 Department of Neurology, University of Ioannina, School of MedicineIoannina, Greece; 4 Second Department of Neurology, University of Athens, School of MedicineAthens, Greece; 5 International Clinical Research Center, St. Anne's University Hospital in BrnoCzech Republic

**Keywords:** Aortic aneurysm, atherosclerosis, coronary artery disease, ischemic stroke, peripheral arterial disease, remote ischemic preconditioning

## Abstract

**Background:**

Remote ischemic preconditioning (RIPC) is the application of a transient and brief ischemic stimulus to a distant site from the organ or tissue that is afterward exposed to injury ischemia, and has been found to reduce ischemia–reperfusion injury (IRI) in various animal models. RIPC appears to offer two distinct phases of endothelial IRI protection, which are presumably mediated through neuronal and humoral pathways.

**Methods:**

We conducted a comprehensive literature review on the available published data about the potential effect of RIPC in patients undergoing IRI in one or more vital organs.

**Results:**

Our search highlighted 24 randomized clinical trials about the effect of RIPC on variable clinical settings (abdominal aortic aneurysm repair, open heart surgery, percutaneous coronary intervention, living donor renal transplantation, coronary angiography, elective decompression surgery, carotid endarterectomy, recent stroke, or transient ischemic attack combined with intracranial carotid artery stenosis). Most of the trials focused on postoperative cardiac or renal function after RIPC with conflicting results. Preconditioning protocols, age limits, comorbidities, and concomitant drug use varied significantly across trials, and therefore no firm conclusions can be drawn using the available data. However, no severe local adverse events were observed in any patient undergoing limb or arm preconditioning.

**Conclusions:**

RIPC is a safe and well-tolerated procedure that may constitute a potentially promising innovative treatment in atherosclerotic diseases. Large, multicenter, randomized clinical trials are required to determine an optimal protocol for the RIPC procedure, and to evaluate further the potential benefits of RIPC in human ischemic injury.

## Introduction

Transient, brief periods of ischemia are considered to trigger pathways that confer protection against a subsequent, more prolonged ischemia in the same tissue. This phenomenon is known as ischemic preconditioning (IPC). When the precedent ischemic stimulus is applied to a distant site from the organ or tissue that is afterward exposed to injury ischemia, the preconditioning is remote, and thus the procedure is named as remote ischemic preconditioning (RIPC) (Veighey and Macallister 2012). RIPC has been described to reduce ischemia–reperfusion injury (IRI) in various animal models. The promising results from animal studies raised expectations that preconditioning could provide the analogous benefits in patients with various tissue ischemia injuries, and thus RIPC protocols were transferred and further tested in numerous clinical trials (Lazaris et al. 2009).

In view of the former considerations we conducted a comprehensive narrative review regarding the available clinical data on the safety and efficacy of RIPC in the treatment of atherosclerotic diseases.

## Methods

We systematically reviewed published data about the potential effect of RIPC in the postprocedural outcome of patients undergoing IRI in one or more vital organs. Our literature search through MEDLINE and EMBASE was based on the term “remote ischemic preconditioning” and was focused on human studies. Last search has been performed on 14 May 2013. References of retrieved articles were also screened. Reference lists of all articles that met the criteria and of relevant review articles were examined to identify studies that may have been missed by the database search. Duplicate publications and articles not written in English language were excluded from further evaluation.

## Results

### Potential mechanisms of action of RIPC

Remote ischemic preconditioning appears to offer two distinct phases of endothelial IRI protection in humans, both of which are mediated from the autonomic nervous system. The early, short phase is activated immediately after preconditioning and vanishes within 4 h, whereas the second, prolonged phase presents 24 h after the preconditioning stimulus and lasts for at least 48 h (Kharbanda et al. 2002; Loukogeorgakis et al. 2005).

Not only neuronal signaling but also several humoral mediators and diverse humoral pathways (opioids, nitric oxide (NO), adenosine, bradykinin, catecholamines, heat-shock proteins, heme oxygenase, tumor necrosis factor a (TNF-a), angiotensin, and prostaglandins) have been suggested to have a key role in the transduction of the preconditioning stimulus to the remote tissue that is exposed to ischemic injury (Kanoria et al. 2007a). According to Lang et al. (2006), the mediator that transfuses the RIPC stimulus should be a protein with a molecular weight of no more than 8 kDa.

Numerous studies unveiled an activation of opioid receptors as a regulatory mechanism in tissues that have been exposed to reperfusion ischemia injury, suggesting that endogenous opioids can confer both acute and chronic ischemic protection (Peart et al. 2005). NO, a known major adenosine-induced vasodilator, has also been associated with the protective effects of preconditioning (Teoh 2011). Apart from locally induced vasodilation, NO may trigger other signal pathways and induce hepatic heme oxygenase-1 (HO-1), a stress inducible protein with antiinflammatory effects (Kanoria et al. 2007b; Lai et al. 2007). However, NO synthase inhibition has been unable to abolish the preconditioning-induced protection, suggesting that NO generation may not be the sole mechanism of RIPC (Petrishchev et al. 2001). Hydrogen sulfide (H_2_S), a metabolite generated by cells under conditions of ischemia, has similar properties with NO (vasodilation, antiinflammatory properties, heme proteins induction, mitochondrial redox signaling, and K_ATP_ channels opening) and thus could be another possible mediator of the RIPC stimulus (Osswald and Moerike 2011). Mitochondrial K_ATP_ channels are thought to be a plausible target of the RIPC, whose significance is further portrayed in a case of heart transplantation by Kristiansen et al. (2005). Compared with controls, preconditioning groups were found to have increased heat-shock protein 70 (HSP 70) levels in myocardial tissue and serum inflammatory mediators (IL-6, IL-8, IL-10, and TNF-a) (Zhou et al. 2010; Li et al. 2013). However, in human leukocytes, the RIPC stimulus was found to suppress proinflammatory gene transcription while upregulating both heat-shock proteins and calpastatin (Konstantinov et al. 2004). Finally, results from both cardiac and kidney ischemia models suggest that mitogen-activated protein kinase pathways might also have a significant role in the preconditioning-induced protection from ischemia (Park et al. 2001; Heidbreder et al. 2008).

### RIPC in clinical trials of patients undergoing abdominal aortic aneurysm repair

Table 1 summarizes the design and results of four randomized clinical trials evaluating the safety and efficacy of RIPC in patients undergoing abdominal aortic aneurysm repair. In a very recent randomized clinical trial of 62 patients by Li et al., three cycles of 5-min upper left-arm ischemia followed by 5-min reperfusion after anesthesia induction were found to diminish both pulmonary injury (assessed by alveolar:arterial oxygen tension ratio), intestinal injury (assessed by serum intestinal fatty acid–binding protein, endotoxin levels, and diamine oxidase activity), and systemic inflammatory response in the first 24 h (assessed by IL-6 and TNF-a) after elective open abdominal aortic aneurysm repair (Li et al. 2013).

**Table 1:** Safety and efficacy of remote ischemic preconditioning (RIPC) in randomized clinical trials (RCTs) of abdominal aortic aneurysm repair (AAA)

Author	No. patients	Study type	Intervention	RIPC protocol	Cuff pressure	Limp	RIPC time	Adverse events	Results
Ali et al. (2007)	82	RCT	Elective open AAA repair	2 × (10-min intermittent cross-clamping/10-min reperfusion)	–	Iliac artery	Before opening of the aneurysm sac	–	RIPC decreased postoperative myocardial injury or infarction and renal impairment
Li et al. (2013)	62	RCT	Elective open AAA repair	3 × (5-min ischemia/5-min reperfusion)	200 mmHg	Arm	After anesthesia induction	No	RIPC diminished pulmonary and intestinal injury in the first 24-h postoperatively
Walsh et al. (2009)	40	RCT	Endovascular AAA repair	2 × (10-min alternate ischemia)	Until no Doppler signal	Leg	After anesthesia induction	No	RIPC lowered the biomarkers of renal injury
Walsh et al. (2010a,b)	40	RCT	Elective open AAA repair	2 × (10-min intermittent iliac artery cross-clamping/10-min reperfusion	–	Iliac artery	Before opening of the aneurysm sac	3 deaths and 4 cases of lower limp ischemia in the RIPC group	RIPC had no effect on postoperative renal injury

In another double-blind randomized control trial of 82 patients by Ali et al. (2007), preconditioned patients undergoing elective open abdominal aortic aneurysm repair were found to have lower rates of postoperative myocardial injury (assessed by cardiac troponin I release – TnI > 0.40 μmol/mL), myocardial infarction, and renal impairment (assessed by serum creatinine >177 μmol/L) compared with controls (27, 22, and 23%, respectively). The ischemic stimulus was delivered during the operation, just before the opening of the aneurysm, and consisted of two cycles of intermittent cross-clamping of the common iliac artery for 10 min followed by 10 min of reperfusion (Ali et al. 2007). Following the aforementioned protocol, a small randomized control trial by Walsh et al. (2010a,b) did not reveal any significant effect of RIPC on renal injury, assessed with both urinary retinol-binding protein and albumin:creatinine ratio, following elective open abdominal aortic aneurysm repair. Of note is that only in the preconditioned group three patients died of cardiac or embolic causes and four patients developed lower limp ischemia requiring intervention (Walsh et al. 2010b).

In endovascular abdominal aortic aneurysm repair of 40 male patients, biomarkers of renal injury (urinary retinol-binding protein and urinary albumin:creatinine ratio) were lower in patients who had two sequential 10-min periods of alternate lower limb ischemia immediately after induction of anesthesia and urinary catheterization. However, the rates of adverse major cardiac outcomes, renal impairment, and serum troponin elevation were similar between the preconditioned and control groups (Walsh et al. 2009).

### RIPC in clinical trials of patients undergoing open cardiac surgery

Table 2 summarizes the design and results of 13 randomized clinical trials evaluating the safety and efficacy of RIPC in patients undergoing open cardiac surgery. Findings from a randomized clinical trial of 60 infants by Zhou et al. (2010) support that limb RIPC is not only safe to apply in infants, but can also ameliorate systemic inflammatory response and protect against myocardial and pulmonary IRI after open heart surgery supported by cardiopulmonary bypass. The preconditioning protocol consisted of three cycles of 5-min limb ischemia followed by 5-min reperfusion, 24 and 1 h before the start of the surgery (Zhou et al. 2010).

**Table 2:** Safety and efficacy of remote ischemic preconditioning (RIPC) in randomized clinical trials (RCTs) of open heart surgery

Author	No. Patients	Study type	Intervention	RIPC protocol	Cuff pressure	Limp	RIPC time	Adverse events	Results
Cheung et al. (2006)	37	RCT	Open heart surgery	4x(5-min ischemia/5-min reperfusion)	SBP +15 mmHg	Leg	5–10 min before the initiation of CPB	–	RIPC reduced postoperative myocardial and lung injury
Choi et al. (2011)	76	RCT	Complex valvular surgery	3 × (10-min ischemia/10-min reperfusion	200 mmHg	Leg	5–10 min before the initiation of CPB	No	RIPC was not associated with lower AKI incidence or lower renal biomarkers
Gunaydin et al. (2000)	8	RCT	CABG	2 × (3-min ischemia/2-min reperfusion)	300 mmHg	Arm	After CPB	No	RIPC was related to lower postoperative LDH levels, but not significant differences in both CPK or CK-MB levels
Hausenloy et al. (2007)	57	RCT	CABG	3 × (5-min ischemia/5-min reperfusion)	200 mmHg	Arm	After anesthesia induction	–	RIPC reduced perioperative serum cTnT levels
Hong et al. (2010)	65	RCT	CABG	4 × (5-min ischemia/reperfusion)	–	Arm	Before aortic clamping	–	RIPC reduced, although not significantly, postoperative cTnT levels
Li et al. (2010)	81	RCT	Elective valve replacement	3 × (4-min ischemia/4-min reperfusion)	600 mmHg	Leg	After anesthesia induction or after aortic cross-clamping	No	RIPC only after aortic cross-clamping was related to lower postoperative cTnT levels
Pedersen et al. (2012)	105	RCT	Open heart surgery	4 × (5-min ischemia/5-min reperfusion)	SBP +40 mmHg	Leg	Before surgery	–	RIPC was not associated with lower AKI or lower renal biomarkers
Rahman et al. (2010)	162	RCT	CABG	3 × (5-min ischemia/5-min reperfusion)	200 mmHg	Arm	Before aortic clamping	–	RIPC was not related to neither postoperative cTnT release, blood hemodynamics, renal dysfunction, lung injury or total hospital/ICU stay
Thielmann et al. (2010)	53	RCT	CABG	3 × (5-min ischemia/5-min reperfusion)	200 mmHg	Leg	After anesthesia induction	–	RIPC decreased both postoperative mean cTnT and serum creatinine levels
Venugopal et al. (2009)	45	RCT	CABG	3 × (5-min ischemia/5-min reperfusion)	200 mmHg	Arm	After anesthesia induction	–	RIPC reduced perioperative serum cTnT levels
Venugopal et al. (2010)	78	Secondary analysis of 2 RCT	CABG	3 × (5-min ischemia/5-min reperfusion)	200 mmHg	Arm	After anesthesia induction	–	RIPC decreased the postoperative incidence of AKI and perioperative cTnT release
Zhou et al. (2010)	60	RCT	Open heart surgery	3 × (5-min ischemia/5-min reperfusion)	240 mmHg	Arm	24-h and 1-h before surgery	No	RIPC protected against myocardial and pulmonary injury
Zimmerman et al. (2011)	120	RCT	Elective cardiac surgery	3 × (5-min ischemia/5-min reperfusion)	200 mmHg	Leg	After anesthesia induction	–	RIPC reduced the postoperative AKI incidence

SBP, systolic blood pressure; CABG, coronary artery bypass grafting; CPB, cardiopulmonary bypass; AKI, acute kidney injury; cTnT, cardiac troponin T; CPK, creatinine phosphokinase; CK-MB, creatinine kinase MB).

Similarly, preconditioned children with congenital heart defects, undergoing four cycles of 5-min lower limp ischemia followed by 5-min reperfusion before cardiopulmonary bypass and subsequent cardiac surgery, had postoperatively lower rates of myocardial (troponin T release) and lung injury (airway resistance) (Cheung et al. 2006). In another randomized clinical trial of 105 children undergoing congenital heart defects surgery by Pedersen et al. (2012), preconditioning using the aforementioned protocol was neither related to lower incidence of postoperative acute kidney injury (defined by serum creatinine and urinary output) nor with significant changes in more recently developed renal biomarkers including plasma and urinary neutrophil gelatinase–associated lipocalin (NGAL) and plasma cystatin C. However, it should be noted that the study by Pedersen et al. (2012) was underpowered to detect a reduction in acute kidney injury less than 30% between the preconditioned and control group. Moreover, a subanalysis of that study revealed that patients over 6 months of age benefited from RIPC (Pedersen et al. 2012; Tweddell 2012).

In another study protocol of 76 adult patients undergoing complex valvular heart surgery by Choi et al. (2011), no significant differences in the incidence of postoperative acute kidney injury and the concentrations of serum creatinine, cystatin, or NGAL were noticed between controls and patients preconditioned with three cycles of 10-min lower limb ischemia followed by 10-min reperfusion. However, preconditioning was related to both lower (creatine kinase MB) CK-MB levels 24 h postoperatively and shorter intensive care unit (ICU) stay (Choi et al. 2011). In a randomized control trial of 81 patients undergoing elective valve replacement by Li et al. (2010), preconditioning with three cycles of 4-min lower limb ischemia followed by 4-min reperfusion after anesthesia induction had no effect on serum troponin T release. Interestingly, the other group of patients who received the aforementioned preconditioning stimulus immediately after aortic cross-clamping had significantly lower (40%) postoperative troponin T levels compared with the control group (Li et al. 2010).

### RIPC in clinical trials of patients undergoing cardiac bypass graft surgery

In a preliminary study of eight male patients undergoing coronary artery bypass graft surgery (CABG) by Gunaydin et al. (2000), preconditioning with two cycles of a 3-min right-arm ischemia followed by 2-min reperfusion was related to only lower lactate dehydrogenase (LDH) levels 5-min after clamping the aorta compared with controls. No significant perioperative or postoperative differences in (creatine phosphokinase) CPK or CK-MB levels between the two groups were noted (Gunaydin et al. 2000). Preconditioning with three cycles of 5-min right upper limb ischemia followed by 5-min reperfusion before CABG was related to reduced perioperative serum troponin T levels in two independent randomized clinical trials of 57 patients by Hausenloy et al. (2007) and 45 patients by Venugopal et al. (2009). A secondary analysis of these two randomized trials revealed a postoperative decrease in the incidence of acute kidney injury in nondiabetic preconditioned patients after CABG compared with controls (Venugopal et al. 2010).

Thielmann et al. (2010) used the same preconditioning protocol in a single-blind, randomized clinical trial of 53 nondiabetic patients with triple-vessel disease who underwent CABG with crystalloid cardioplegic arrest. They found both a significant decrease in mean troponin T release (44.5%) and peak serum creatinine concentration postoperatively in the preconditioned group when compared with controls (Thielmann et al. 2010). Hong et al. (2010) found a 26% total reduction in postoperative troponin T in 65 patients preconditioned with four cycles of 5-min upper limb ischemia followed by reperfusion that underwent off-pump CABG, when compared with controls. However, this decrease did not reach statistical significance (Hong et al. 2010).

In a single-blind, randomized clinical trial of 120 patients undergoing elective cardiac surgery (CABG, valve surgery, combined, or other), Zimmerman et al. (2011) found that preconditioning (three cycles of 5-min limb ischemia followed by 5-min reperfusion) decreased the incidence of acute kidney injury within 48 h after surgery by 27%; even though a history of previous heart surgery – a known risk factor for acute kidney injury – was significantly more common in control patients compared with the preconditioned group. Using the aforementioned preconditioning stimulus in a larger, randomized clinical trial of 162 patients undergoing coronary artery bypass surgery, Rahman et al. (2010) found no correlation of RIPC with troponin release, blood hemodynamics, renal dysfunction, lung injury, or total hospital/ICU stay. However, it should be taken into consideration that patients with angina or with an acute coronary syndrome within 30 days of surgery were not excluded in this study protocol by Rahman et al. (2010).

### RIPC in clinical trials of patients undergoing percutaneous coronary intervention for acute myocardial infarction

Table 3 summarizes the design and results of five randomized clinical trials evaluating the safety and efficacy of RIPC in patients undergoing percutaneous coronary intervention (PCI) for acute myocardial infarction. In a randomized clinical trial of 41 consecutive patients with stable angina and single-vessel disease undergoing PCI and stent implantation, Iliodromitis et al. (2006) found that preconditioned patients with three cycles of 5-min upper limb ischemia followed by 5-min reperfusion had significantly higher troponin T and CK-MB levels 24 h after the intervention, when compared with controls. Interestingly, a milder rise of cardiac enzymes was observed in the subgroup of preconditioned patients who were on statin treatment, suggesting that statins may ameliorate the inflammatory response after preconditioning (Iliodromitis et al. 2006).

**Table 3:** Safety and efficacy of remote ischemic preconditioning (RIPC) in randomized clinical trials (RCTs) of percutaneous coronary intervention (PCI) for the treatment of myocardial infarction (MI)

Author	No. Patients	Study type	Intervention	RIPC protocol	Cuff pressure	Limp	RIPC time	Adverse events	Results
Botker et al. (2010)	333	RCT	PCI	4 × (5-min ischemia/5-min reperfusion	200 mmHg	Arm	During ambulance transfer	–	RIPC was related to greater myocardial salvage, mainly in patients with total vessel occlusion or LAD infarction
Hoole et al. (2009a)	242	RCT	PCI	3 × (5-min ischemia/5-min reperfusion	200 mmHg	Arm	Before PCI	No	RIPC was related to less perioperative chest discomfort and ST deviation, with lower postoperative cTnT release at 24-h and less adverse vascular events at 6 months
Hoole et al. ([Bibr b14][Bibr b15])	40	RCT	PCI	3 × (5-min ischemia/5-min reperfusion	200 mmHg	Arm	Before PCI	–	RIPC had no effect on LV dysfunction during PCI
Iliodromitis et al. (2006)	41	RCT	PCI	3 × (5-min ischemia/5-min reperfusion	200 mmHg	Both Arms	Before PCI	–	RIPC was related to higher postoperative cTnT and CK-MB levels, and this effect was milder in patients treated with statins
Munk et al. (2010)	232	RCT	PCI	4 × (5-min ischemia/5-min reperfusion	200 mmHg or SBP+25 mmHg if SBP>175 mmHg	Arm	During ambulance transfer	–	RIPC significantly improved LV function in patients with large myocardial area-at-risk or LAD infarction

SBP, systolic blood pressure; LV, left ventricle; cTnT, cardiac troponin T; LAD, left anterior descending artery; CPK, creatinine phosphokinase; CK-MB, creatinine kinase MB).

Using the same preconditioning protocol in a larger randomized clinical trial of 242 patients undergoing PCI, Hoole et al. (2009a) found that preconditioned patients experienced less chest discomfort and had lower electrocardiographic ST-segment deviation during stent implantation compared with controls. Moreover, preconditioning was both related to lower median troponin T release at 24 h and less cardiac or cerebral adverse events at 6 months (Hoole et al. 2009a). Even though when Hoole et al. (2009b) applied the same preconditioning protocol in 20 patients with single-vessel disease, it was not found to ameliorate left ventricular dysfunction during PCI compared with 20 controls. A possible explanation of the lack of effect of preconditioning on left ventricular dysfunction during PCI could be that single-vessel coronary disease may not be sufficient to induce a significant reperfusion injury (Hoole et al. 2009b), and thus this hypothesis needs to be retested in patients with more severe coronary disease.

Subsequently, Munk et al. (2010) in their randomized clinical trial of 232 patients with first (ST elevation myocardial infarction) STEMI found that left ventricular function was significantly improved in preconditioned patients with large myocardial area-at-risk and/or left anterior descending artery infarcts. However, it should be noted that in the study by Munk et al. (2010), preconditioning stimulus was offered in patients during ambulance transfer and consisted of four cycles of 5-min upper limb ischemia followed by 5-min reperfusion. The same preconditioning protocol during ambulance transfer has also been used by Botker et al. (2010) in their randomized clinical trial of 333 consecutive patients with first acute myocardial infarction. They found that preconditioned patients had a greater myocardial salvage, assessed with single-photon emission CT (SPECT), after PCI compared with controls and this effect was more robust in patients with totally occluded vessels and infarcts in the left anterior descending artery. However, left ventricular ejection fraction at 30 days and troponin T release 90–102 h after angioplasty were not significantly different between the preconditioned and control groups (Botker et al. 2010).

### RIPC for the protection against contrast medium-induced acute kidney injury and reperfusion ischemia injury in renal transplantation

Preconditioning with four cycles of 5-min upper arm ischemia followed by 5-min reperfusion was found to ameliorate contrast medium-induced kidney injury after elective coronary angiography in a randomized control trial of 100 patients with primarily impaired renal function (Er et al. 2012). In a recent randomized clinical trial of patients undergoing living donor renal transplantation, neither donor nor recipient preconditioning with three cycles of 5-min upper limb ischemia followed by 5-min reperfusion had an effect on renal function and biochemical markers within 72 h after transplantation, or even on the duration of total hospital stay (Chen et al. 2013).

### RIPC in clinical trials of extracranial or intracranial atherosclerosis

Table 4 summarizes the design and results of two randomized clinical trials evaluating the safety and efficacy of RIPC in patients with extra- or intracranial atherosclerosis. In a pilot randomized clinical trial of 70 patients undergoing carotid endarterectomy, preconditioned patients with two cycles of alternate 10-min lower limb ischemia followed by reperfusion after anesthesia induction were found to have lower rates of sustained intraoperative hypotension compared with controls. However, the study was underpowered to reveal any differences in the neurological outcome between preconditioned and control group (Walsh et al. 2010a). Meng et al. (2012) conducted a randomized clinical trial of 68 Chinese patients with a recent history of stroke or transient ischemic attack (TIA) and simultaneous intracranial arterial stenosis. Preconditioned patients who had five cycles of 5-min bilateral upper limb ischemia followed by 5-min reperfusion twice a day for 300 consecutive days had reduced TIA recurrence, increased recovery rate (measured with the modified Rankin scale), and augmented cerebral perfusion (measured with SPECT and transcranial Doppler sonography) (Meng et al. 2012).

**Table 4:** Safety and efficacy of remote ischemic preconditioning (RIPC) in randomized clinical trials (RCTs) of extracranial or intracranial atherosclerosis

Author	No. Patients	Study type	Intervention	RIPC protocol	Cuff pressure	Limp	RIPC time	Adverse events	Results
Meng et al. (2012)	68	RCT	Patients with symptomatic intracranial stenosis	5 × (5-min ischemia) twice daily	200 mmHg	Both legs	300 consecutive days	No	RIPC reduced TIA recurrence, increased recovery rate and augmented cerebral perfusion
Walsh et al. (2010a,b)	70	RCT	CEA	2 × (10-min alternate ischemia/reperfusion)	Until no Doppler signal	Legs	After anesthesia induction	No	RIPC related to lower rates of intraoperative hypotension

SBP, systolic blood pressure; TIA, transient ischemic attack; ICA, internal carotid artery; CEA, carotid endarterectomy.

## Discussion

We reviewed a total of 24 randomized clinical trials evaluating the safety and efficacy of RIPC in different atherosclerotic diseases including abdominal aortic aneurysm repair (Table 1), open heart surgery (Table 2), PCI (Table 3), and intracranial or extracranial atherosclerosis (Table 4). All studies were either single- or double-blinded RCTs, with the exception of a secondary analysis of two RCTs (Venugopal et al. 2010). Our findings indicate that an optimal protocol for the induc tion of RIPC has not been established yet. Thus, RIPC protocols (including ischemia–reperfusion sequences, cuff pressures, limb choice, and RIPC time) vary significantly among trials (Tables 4). Likewise, the potential effects of age, race, drugs, and comorbidity on RIPC response have not been adequately investigated in the conducted studies so far (Endre 2011).

In the study by Walsh et al. (2009) more preconditioned patients were treated with statins compared with controls. Statin use was found to ameliorate postoperative cardiac enzyme release in the study protocol by Iliodromitis et al. (2006), suggesting that the prevention of both cardiac and renal injury after vascular surgery may be due to the suppression of postprocedural inflammatory response by preoperative statin treatment (Iliodromitis et al. 2006; van Kuijk et al. 2009). Patients older than 80 years of age or patients with diabetes were excluded by study design in many clinical trials (Ali et al. 2007; Hausenloy et al. 2007; Hoole et al. 2009b; Venugopal et al. 2009, 2010; Rahman et al. 2010; Thielmann et al. 2010; Choi et al. 2011). A subgroup analysis in the study by Pedersen et al. (2012) suggests that age stratification might have an important role in the selection of patients who should undergo RIPC procedures (Pedersen et al. 2012; Tweddell 2012), and this potential confounder should be seriously taken into account when interpreting the available trial data.

In all trials, no severe local adverse events were observed, except in the study by Walsh et al. (2009) with iliac cross-clamping, in which three patients died (asystole, myocardial infarction, and cardiac arrest) and four patients developed lower limb ischemia requiring intervention. Minor local adverse events occurred in the study by Cai et al., with slight skin erythema developing in two patients and a temporally constriction feeling in one patient after RIPC (Li et al. 2013). In addition, a phase Ib study of 33 patients by Koch et al. (2011) confirmed that RIPC with limb ischemia is feasible, safe, and well tolerated in alert patients with subarachnoid hemorrhage. Therefore, we may hypothesize that RIPC protocols with limb ischemia are potentially safe and hence can be tested with safety in larger scale randomized clinical trials.

Most of the trials focused on postoperative cardiac and/or renal function after RIPC with conflicting results (Tables 4). Preconditioned patients undergoing abdominal aneurysm artery repair were found to have lower rates of renal injury when compared with controls in a metanalysis by Alreja et al. (2012). In the same metanalysis, RIPC was related to lower levels of postoperative myocardial injury, although the results from the trials that were analyzed were highly heterogeneous (Alreja et al. 2012). In another metanalysis of randomized clinical trials, Pilcher et al. (2012) found that 12 h after open cardiac surgery, RIPC subgroups had significantly lower troponin levels compared with controls. However, there is uncertainty regarding the correctness of the aforementioned result due to the statistical heterogeneity between the studies, as the effect of RIPC on postoperative troponin concentration was significantly milder in fully blinded studies, compared with partially blinded (Pilcher et al. 2012). Similarly, in a metanalysis by Brevoord et al. (2012), troponin release and the incidence of periprocedural myocardial infarction were both significantly decreased in preconditioned patients undergoing cardiac surgery, PCI, or vascular surgery. However, no difference in mortality rates or major adverse cardiovascular events has been found between RIPC subgroup and controls (Brevoord et al. 2012). Also, no difference in mortality rates between the two aforementioned groups has been found in another metanalysis by Desai et al. (2011), and their only significant difference – although not consisted across all trials – was limited in the risk of myocardial infarction, which was more reduced in the RIPC group.

The protective effect of RIPC appears to increase in patients with acute myocardial infarction undergoing PCI (Botker et al. 2010; Munk et al. 2010). The effect of RIPC in patients with non-ST elevation myocardial infarction or unstable angina undergoing urgent PCI needs to be determined in future clinical trials. Additionally, RIPC protocols need to be tested in high-risk surgical patients, to examine if the potential effects of preconditioning will be further amplified (Hausenloy et al. 2007). The RICO trial, a large multicenter RCT to determine the effect of preconditioning on atrial fibrillation and other outcomes following CABG, is already on the way (Brevoord et al. 2011). Finally, other future clinical trials can examine the effect of RIPC during ambulance transfer in patients with acute ischemic stroke or acute myocardial infarction, a practice which not only might salvage valuable ischemic tissue but may also prolong therapeutic window for thrombolysis.

In conclusion, RIPC seems to be an inexpensive, safe, and well-tolerated procedure that ameliorates IRI in remote organs. Potential protective effects of RIPC on different clinical settings (various procedures, age limits, and comorbidities), as well as an optimal protocol for the procedure, need to be further determined in large-scale multicenter RCTs.
